# The Primacy of Cognition in the Manifestations of Substance Use Disorders

**DOI:** 10.3389/fneur.2013.00189

**Published:** 2013-11-18

**Authors:** Jean Lud Cadet, Veronica Bisagno

**Affiliations:** ^1^NIDA Intramural Program, Molecular Neuropsychiatry Research Branch, Baltimore, MD, USA; ^2^Instituto de Investigaciones Farmacológicas (ININFA-UBA-CONICET), Buenos Aires, Argentina

**Keywords:** substance abuse, cognition, impulsivity and self-control, pre-existing condition, frontal cortex

## Introduction

Drug addiction is a serious public health problem that consists of a compulsive drive to take drugs despite repeated severe adverse consequences (1). Factors that influence the development and maintenance of addiction include access to drugs, social environment, genetic predisposition, and psychiatric comorbidities (2). Even in the absence of specific psychiatric diagnoses, certain psychological vulnerabilities may serve as substrates compounding the initiation of drug use and the development of substance use disorders. For example, individual who are sensation-seekers, impulsive, or behavioral disinhibited appear more prone to develop addiction to both licit and illicit substances (3–6). In this context, it is to be noted that not all individuals who try these drugs become addicted as about only 20% of people who have tried drugs become addicts (7). It is also worth mentioning that repeated exposure to moderate to large doses of some of these illicit drugs may be associated with well-known neuropathological consequences (8, 9) that might not be either necessary or sufficient for the development and the maintenance of addicted states.

The accumulated evidence supports the view that a large number of substance users suffer from significant neuropsychological impairments (10). Neuroimaging studies in drug-dependent individuals have also documented significant functional and structural alterations in several brain regions (1). These regions include mesocortical, mesolimbic, and mesostriatal brain regions that are known to be impacted by administration of licit and illicit drugs in both clinical and preclinical studies (2). In what follows, we discuss the potential impact of illicit drugs on these brain regions and the associated cognitive consequences of these drugs. We then suggest that these cognitive consequences play primary roles in the maintenance of addiction across several classes of abused substances.

## Pre-Existing Cognitive Deficits in Substance Use Disorders

Before elaborating the idea of drug-induced cognitive changes in patients addicted to illicit drugs, it is important to briefly discuss the influence of potential premorbid deficits on the cognitive performance of some drugs abusers (11). For example, Ersche et al. (12) suggested that cognitive dysfunctions and impulsive personality traits are endophenotypes for drug dependence. Drug-dependent individuals were significantly impaired on all the tests used in their study and some of these cognitive deficits were also found to exist in first-degree relatives in the absence of drug abuse (12). Levels of impulsivity were also higher in drug-dependent individuals than in their siblings, indicating that chronic drug abuse further increases both anxiety and impulsive traits. Smith et al. (13) also reported that impairments in hemispheric lateralization during task performance were apparent in both drug-using subjects and their control siblings, with the siblings decreasing but the drug users increasing activity in relevant brain regions.

## Addiction as a Hyperconnection Syndrome

The prefrontal cortex (PFC) and the striatum participate in integrated functions that are modulated by glutamate (12) and dopamine (DA) (14). These functions include decision making (15), salience attribution and goal-directed behaviors (16), and inhibitory control of behaviors (17) that are subsumed by specific cortical subregions. Under normal circumstances, organisms consider many salient behaviors but must choose to perform one or the other by performing specific very fast calculations of cost/benefit ratios (18). These activities, including selection of actions based on their valuation, are thought to depend on the PFC (18). Nevertheless, successful completion of goal-directed behaviors such as searching for food or drugs must require more complex and extended sequences of actions that must be maintained despite obstacles and distractions. In people addicted to drugs, there seems to be a narrowing of goal selection, with substances of abuse becoming more salient than other choices (19). This narrowing might be dependent on abnormal dynamics of DA release in the PFC with subsequent effects on corticostriatal glutamate projections to the dorsal striatum that might be intimately involved in the compulsive manifestations of drug-taking behaviors (20, 21). In other words, although the initial effects of drugs might be to enhance DA release in various brain regions (22), repeated exposure to these drugs might lead to subsequent weaker DA release, tolerance to drug effects, followed by increased drug taking to recalibrate the functional interactions of the corticostriatal loops.

Pathological changes in the orbitofrontal cortex (OFC) might also be involved in the manifestation of addiction-related behaviors because it is relevant to outcomes related to primary reinforcers (23). OFC neurons encode details concerning the sensory properties of rewards, the size, and timing of past or future rewards (23). Impairments in the OFC result in compulsive behaviors and impulsivity (24). A potential role of the OFC in the maintenance of addiction is supported by the observation of loss of prefrontal gray matter in individuals with a long history of drug abuse (25). This discussion is also supported by the report of decreased striatal D2R availability, known to be located on the indirect striatal projecting neurons, in the striatum of cocaine and methamphetamine addicts (26, 27). It is thus not farfetched to suggest that unopposed actions of D1-like DA receptors through the direct basal ganglia pathway may promote states of hyperconnections within basal ganglionic/cortical functional and structural loops. These hypothesized hyperconnections might be responsible for several cognitive manifestations of addictive states.

## Manifestations of the Hyperconnection Syndrome

### Impulsivity

Impaired self-control plays a fundamental role in drug-taking behaviors in addictive states (11). Impulsivity is often referred by the term “disinhibition,” referring to the idea of top-down control mechanisms that suppress automatic or reward-driven responses (28). The early stages of recreational drug taking are thought to be due to personality characteristics that influence whether or not the individual will try a rewarding substance. As mentioned earlier, only a minority of these individuals become addicted (7). This fact implies that the rewarding effect of drugs is not the main factor in the development and maintenance of addiction (29). These data also suggest that other important factors might drive the impetus to continue to use or increase the quantity of drugs used by those who become addicted. These factors might include specific pre-existing subclinical or clinical cognitive dysfunctions that might have interfered with an individual’s ability to resist drug-taking behaviors that are known to contribute to adverse life consequences.

The available evidence does support the thesis that impulsivity is a vulnerability marker for substance abuse (11, 12). Several studies have demonstrated that children of drug-using parents have elevated impulsivity before drug exposure and that impulsivity indices are strong and reliable predictors of drug initiation and drug-associated problems (11). Importantly, during the interval of drug taking while the addiction threshold might be broached, taking of substantial amounts of drugs might have produced additional changes in the brains of susceptible individuals, with resulting further progression of cognitive impairments that are well documented in several reports (11, 30–33). Of importance to this thesis, cognitive impairments have been shown to be greater in drug-dependent individuals compared with their siblings (12). The proposed drug-induced pathological and/or functional damage might be responsible for the perseverative aspects of drug-taking behaviors that are somewhat akin to the perseveration observed in patients with severe head traumas (34) or in patients with some demented states (35). This similarity might explain, in part, the perseverative taking of drugs when they are no longer reinforcing and/or when drug-taking behaviors are accompanied by severe psychosocial and medical consequences (23).

Interestingly, abnormalities have been identified in frontal networks that subsume poor self-regulation and impulse control in cocaine dependency. Specifically, cocaine users were reported to show stronger connectivity within the perigenual anterior cingulate cortex (ACC) social processing/“mentalizing” network (36). This study is compatible with previous findings of abnormal inhibitory control in cocaine users (37, 38). Of etiological significance, Kelly et al. (39) found increased but lower resting connectivity between the ACC and dorsolateral prefrontal cortex (DLPFC) in children and adolescents, respectively. This relationship was almost non-existent in adults (39). These observations suggest that the connectivity within these regions might be “pruned” with brain maturation during the aging process and that cocaine users might suffer from a regression of these maturational processes because of plastic effects of the drug. This is important because an efficient interaction of ACC and DLPFC is needed for appropriate behavioral control (40). Therefore, the cognitive impulse control disorder observed in addicted patients might be secondary to drug-induced activation of developmental genes that regulate connectivity between various brain regions (41).

### Attention

Attention represents a number of intimate mechanisms that facilitate the filtering, selection, and processing of information (42). In substance users, there is substantial attentional bias toward substance-related cues (43). Attentional bias exists to a greater extent in people with highly compulsive patterns of drug taking (43). Demonstrations of attentional bias for substance-related stimuli among experienced substance users are consistent with the view that classical conditioning is involved in their development because substance-related cues are, by nature, associated with the effects of substances (44). Other studies have also documented drug-related deficits in attentional tests in cocaine addicts (32, 45, 46). Methamphetamine addicts also showed deficits on measures of sustained (47) and spatial (48) attention, with these deficits having been linked to methamphetamine-associated damage to the ACC and insular cortices (47). Thus, damage to these brain regions might play an important role in causing dysfunctions in attentional circuits that are critical to learning and memory processes that are important to remember specific therapeutic interventions, thereby increasing the rate of recidivism in addicted patients.

### Decision making

Poor cognitive performance in areas of risk-taking and decision making may influence the degree to which illicit drug users engage in risky behaviors with consequent negative health consequences. Deficits in tests of decision making have been found in patients who suffer from marijuana (49, 50), cocaine (51), MDMA (52), and methamphetamine (53, 54) addiction. These deficits might be related to altered connectivity of the right insula to the dorsomedial PFC, inferior frontal gyrus, and DLPFC in cocaine-dependent subjects (55). Methamphetamine-dependent individuals also showed disrupted risk-related processing in the ACC and insula (54). Moreover, occasional stimulant users also showed reward dysfunction during reinforcement-based decision making and greater activation in the ACC, inferior frontal gyrus, and dorsal striatum compared with control subjects (56). Therefore, drug addiction might be associated with altered functional connectivity in large-scale neural networks that might serve to distort awareness, salience detection, and other functions that regulate various cognitive processes including decision making, with subsequent severe negative impacts on activities of daily living (55).

## Conclusion

In summary, drug addiction is marked by mild, yet pervasive, cognitive disruptions that may cause the negative progression of the clinical course, threaten sustained abstinence (57), or increase recidivism (58, 59) associated with addiction to licit and illicit substances. Importantly, the existence of cognitive deficits identified in drug-dependent individuals suggests that cognitive disturbances might be predisposing risk factors for the development of drug dependence (12). Repeated exposure to various drugs of abuse might exacerbate some of these subclinical abnormalities by producing pathological changes in various brain regions including the PFC (2). Interestingly, recent neuroimaging studies have also documented cortical abnormalities in patients addicted to various classes of illicit substances (60). These cortical abnormalities, by causing disinhibition of various cortico-cortical, subcortico-midbrain, or subcortico-cortical pathways, might engender the formation of hyperconnected subcortical loops that might serve as substrates for the varied clinical manifestations of addiction. Better understanding of these neural connection-induced cognitive deficits should help to develop better pharmacological and behavioral approaches for the treatment of substance use disorders.
